# Reactive ion etching for fabrication of biofunctional titanium nanostructures

**DOI:** 10.1038/s41598-019-55093-y

**Published:** 2019-12-11

**Authors:** Mahya Ganjian, Khashayar Modaresifar, Hongzhi Zhang, Peter-Leon Hagedoorn, Lidy E. Fratila-Apachitei, Amir A. Zadpoor

**Affiliations:** 1 Department of Biomechanical Engineering, Faculty of Mechanical, Maritime, and Materials Engineering, Delft University of Technology, Mekelweg 2, 2628 CD Delft, The Netherlands; 2 Department of Materials, Mechanics, Management & Design, Faculty of Civil Engineering and Geosciences, Delft University of Technology, Stevinweg 1, 2628 CN Delft, The Netherlands; 3 Department of Biotechnology, Faculty of Applied Sciences, Delft University of Technology, Van der Maasweg 9, 2629 HZ Delft, The Netherlands

**Keywords:** Bioinspired materials, Implants

## Abstract

One of the major problems with the bone implant surfaces after surgery is the competition of host and bacterial cells to adhere to the implant surfaces. To keep the implants safe against implant-associated infections, the implant surface may be decorated with bactericidal nanostructures. Therefore, fabrication of nanostructures on biomaterials is of growing interest. Here, we systematically studied the effects of different processing parameters of inductively coupled plasma reactive ion etching (ICP RIE) on the Ti nanostructures. The resultant Ti surfaces were characterized by using scanning electron microscopy and contact angle measurements. The specimens etched using different chamber pressures were chosen for measurement of the mechanical properties using nanoindentation. The etched surfaces revealed various morphologies, from flat porous structures to relatively rough surfaces consisting of nanopillars with diameters between 26.4 ± 7.0 nm and 76.0 ± 24.4 nm and lengths between 0.5 ± 0.1 μm and 5.2 ± 0.3 μm. The wettability of the surfaces widely varied in the entire range of hydrophilicity. The structures obtained at higher chamber pressure showed enhanced mechanical properties. The bactericidal behavior of selected surfaces was assessed against *Staphylococcus aureus* and *Escherichia coli* bacteria while their cytocompatibility was evaluated with murine preosteoblasts. The findings indicated the potential of such ICP RIE Ti structures to incorporate both bactericidal and osteogenic activity, and pointed out that optimization of the process conditions is essential to maximize these biofunctionalities.

## Introduction

It is known that micro- and nano-scale topographies have a significant impact on the behavior of both eukaryotic and prokaryotic cells^1^. For example, nanowires^2^, nanopillars^3–12^, nanotubes^13^, and nanopits^14,15^ with specific dimensions have been shown to exhibit antibacterial properties. Developing such structures provides a drug-free approach to combat infections which is considered as an alternative to the common antibacterial surfaces which release antibacterial agents^16–18^. The characteristics of such topographies (*e.g*., shape, height/depth, diameter, interspace, and spatial arrangement) are important factors that can be also used to influence the stem cell fate^19^. Therefore, harnessing nanoscale topography represents a powerful approach for achieving the required biofunctionalities of implants, such as improved osseointegration (*i.e*., integration into the host bony tissue) and antibacterial properties. That is why techniques that enable fabrication of controlled topographies on suitable biomaterials are of growing interest.

Several techniques have been used to create topographies which discourage bacterial colonization. Examples include hydrothermal treatment^3,20–24^, reactive ion etching (RIE)^5,8,9,12,25^, pulsed plasma polymerization^26^, electron-beam lithography^14^, anodization^27^, and nanoimprint lithography^10^.

Recent studies demonstrate that bacterial adhesion on nanostructured surfaces is strictly correlated with the nanoscale morphological features of the surface, indicating that the bactericidal effect is highly morphology-dependent^28,29^. Minor changes in the characteristics of these surfaces can therefore have a major impact on their bactericidal efficiency, as demonstrated in a recent study based on black Si (bSi) surfaces^9,12^. For instance, the sharper tip of bSi nanostructures compared with cicada wing and dragonfly wing, as two naturally occurring bactericidal surfaces, leads to a higher killing efficiency against both Gram-positive and Gram-negative bacteria^4^. In another study, the highest killing efficiency was obtained when the interspace was between 130 and 380 nm^10^. Therefore, to harness the potential of nanotopographies for specific biofunctionalities, their morphological features should be precisely controlled and optimized.

RIE can rapidly generate biomimetic, high aspect ratio nanostructures (*e.g*., inspired by the dragonfly wing^4,30,31^, damselfly wing^32^, and gecko skin^13^) on large areas without any need for masks. This process has been applied to create bSi topographies with antibacterial properties^4,5,9,28^. However, silicon is not a proper choice of material for orthopedic implants.

Titanium and its alloys represent one of the most important groups of biomaterials^33^ for orthopedic and dental applications due to their combination of properties such as cytocompatibility, corrosion resistance^34,35^, fatigue properties^34,35^, low density, and relatively low elastic modulus^36–38^ as compared to other metallic biomaterials. Creating RIE topographies on titanium substrates is therefore clinically highly relevant.

Femtosecond laser ablation^33^, hydrothermal treatment^2,20,22,23,39^, and anodizing^13^ are the most commonly used methods to create nanostructures on titanium substrates. Jaggessar *et al*. have studied the effects of different hydrothermal treatment conditions such as process temperature, time, and NaOH concentration on the resulting nanotopographies on Ti, followed by investigations of these surfaces with regard to their mechanical properties and bactericidal effects^22^. Although the hydrothermal method is environmentally friendly, simple, and inexpensive, it is relatively slow as compared to RIE (>2 hr as compared to a few min). The maximum killing efficiency of this work for Gram-positive *Staphylococcus aureus* bacteria is reported to be 54% and 33%, after 3 and 18 hr incubation times, respectively^22^. In another work, which used the hydrothermal technique to create TiO_2_ nanowires on the titanium substrate^20^, the bactericidal effects of the created nanostructures against *S. aureus* were insignificant. This can be explained by the fact that it is generally difficult to produce nanowires with a sharp tip using the hydrothermal technique. Fadeeva *et al*. have reported the bacterial response to superhydrophobic, self-organized titanium microstructures created by femtosecond laser ablation, but the killing efficiency has not been reported^33^, and there are also some limitations in reducing the size of the structures to nanometers.

Recently, Hasan *et al*. have reported that black Ti surfaces produced by inductively coupled plasma reactive ion etching (ICP RIE) can be bactericidal^40^. The killing efficiency of bactericidal nanostructures produced by ICP RIE method is reported to be much higher than the values found when using other processes (95 ± 5% for *E. coli* and 98 ± 2% for *P. aeruginosa* after 4 hr, and 76 ± 4% for *S. aureus* after 20 hr). The shape, dimensions, wettability, and mechanical characteristics of ICP RIE have important effects on the cell response. Yet, the effects of ICP RIE parameters on those characteristics are unknown.

ICP RIE is based on dry chemical etching and physical ion bombardment^41^. The resulting etched profile can be isotropic or anisotropic based on the conditions used^42^. Titanium etching is mostly driven by the chemical process, while TiO_2_ etching relies on physical processes^43^. While chemical process relies on the reaction between the etching gasses and the substrate, physical process is driven by the kinetic energy of particle beams, *i.e*., radical and ion beam to attack the substrate and remove the material^44^. The main difference between RIE and ICP RIE mechanisms is that in RIE, the control over the process is limited and the plasma density is low, which means that higher power is required to have the desired morphology^41^. ICP RIE is a double-powered plasma system in which an RF generator applies power to the ICP coil to control the ion flux and plasma density. Moreover, a bias power is applied to the lower electrode using a 13.56 MHz RF generator to accelerate the ions towards the substrate. Therefore, it is possible to have independent control over the energy of etchant ions and ion current, which makes the process window larger. Taking advantage of this property, ICP RIE is capable of working at low pressures (10^–3^–10^−4^ mbar), while RIE is not stable in this range.

The aim of this work was to investigate the effects of ICP RIE conditions on the morphology, hydrophilicity, and mechanical properties of the resulting structures on titanium surfaces, as a necessary step towards defining their potential to offer desirable surface biofunctionalities for titanium implants. The ICP RIE conditions studied were: time, RF and ICP power, chamber pressure, temperature, and gas composition. Mechanical tests using nanoindentation were also carried out to measure the elastic modulus and hardness of three types of ICP RIE nanoscale structures. Following characterization, the antibacterial properties and cytocompatibility of such ICP RIE treated surfaces were preliminarily assessed by exposing them to both Gram-positive and Gram-negative bacteria using strains that are commonly encountered in implant-associated infections, and by culturing preosteoblast cells.

## Results

### Surface characteristics of the polished titanium

**Figure 1:**
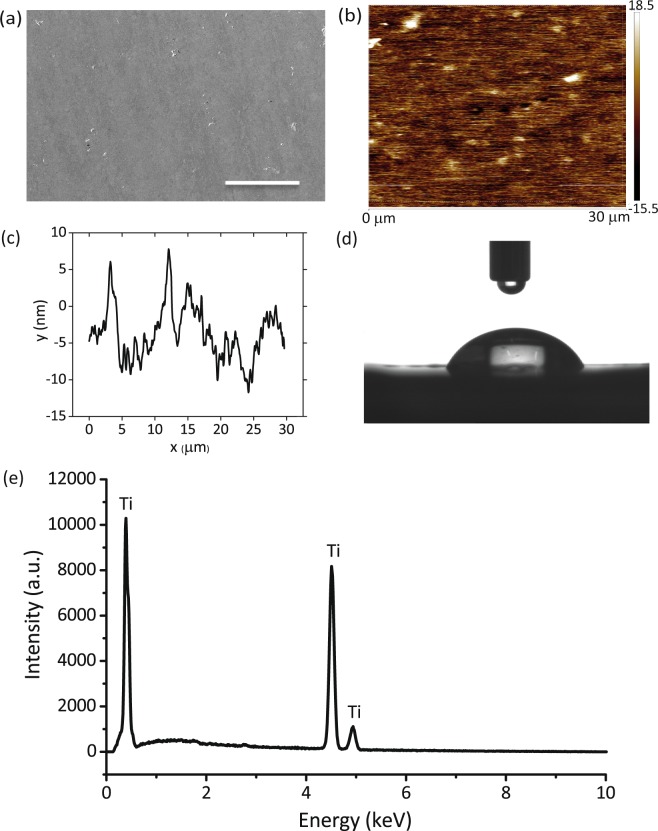
Characterization of the CMP polished titanium surface. (**a**) SEM image of the polished titanium surface. The scale bar is 50 µm. (**b**) AFM image of the polished surface, (**c**) The AFM line profile of the polished surface, (**d**) Photograph of a water droplet on the surface of the polished titanium specimens, (**e**) EDX characterization of the polished titanium surface.

Following polishing, titanium surfaces revealed a very smooth topography with fine polishing lines visible (Fig. 1a,b). *R*_*a*_ and *R*_*q*_ as determined by AFM were 3.79 ± 0.98 nm and 4.93** ± **1.41 nm, respectively (Fig. 1b,c). The water contact angle of the polished surfaces was 70.4 ± 1.8**°** (Fig. 1d). The major component of the polished specimens was titanium (≈94.5%) (Fig. 1e).

### The effects of ICP RIE process time on the resultant Ti surfaces

**Figure 2:**
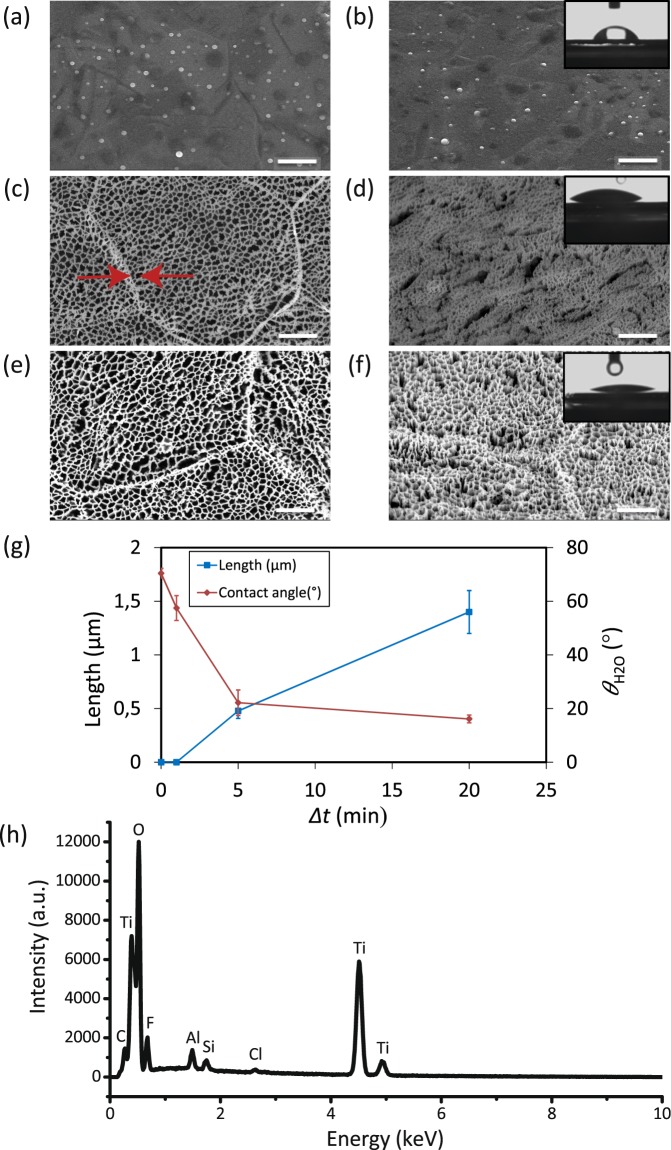
SEM micrographs of the Ti nanostructures created using different values of Δ*t*: (**a,b**) 1 min, (**c,d**) 5 min, and (**e,f**) 20 min. The left sub-figures were taken from the top view while the right sub-figures were taken at 35° tilted view. Other etching conditions: Cl_2_ 30 sccm, Ar 2.5 sccm, *T* 20 °C, *p* 0.02 mbar. *P*_*ICP*_ and *P*_*RF*_ were 600 W and 100 W, respectively. Insets show a droplet of water on the black Ti surface. The scale bars are 1 µm. (**g**) The length and wettability of the Ti nanostructures as a function of Δ*t*. (**h**) EDX characterization of the Ti nanostructures.

One minute of etching was not enough to form any clear etched structure (Fig. 2a,b). Moreover, these specimens showed no change in color following ICP RIE. Five minutes was sufficient to form black Ti nanostructures on the surface (Fig. 2c,d). The very short nanopillars were connected to each other under this condition and formed a porous network (Fig. 2c). In this study, we called the connections between nanopillars “struts” (two red arrows show one of them in Fig. 2c). The well-defined titanium grain boundaries can be seen in Fig. 2c,e. The length of the nanopillars under this condition was 478 ± 70 nm (Fig. 2g, and Table S1) and the thickness of the struts was 25.2 ± 7.5 nm (Table S1).

As Δ*t* was increased from 5 min to 20 min, the length of nanostructures increased to 1.4 ± 0.2 µm (Fig. 2f,g, and Table S1), while the struts of the nanostructures had almost the same thickness as the resultant profile after 5 min etching (Fig. 2e and Table S1).

The surface contact angle of the specimens treated for 1 min decreased from 70.4 ± 1.8° (polished titanium) to 57.5 ± 4.6° (Fig. 2g). Increasing Δ*t* to 5 and 20 min led to porous structures with increased pore sizes (compare Fig. 2c with e), which allowed the surface to be totally wet (insets of Fig. 2d,f). Therefore, these surfaces showed enhanced hydrophilicity, with contact angles of 22.2 ± 4.8° after 5 min and 16.1 ± 1.4° after 20 min etching (insets of Fig. 2d,f, as well as Fig. 2g). The elements present on the surface of black Ti were O (≈ 50%) and Ti (≈ 33%) as well as minor amounts of Al (Fig. 2h).

### The effects of RF power

**Figure 3:**
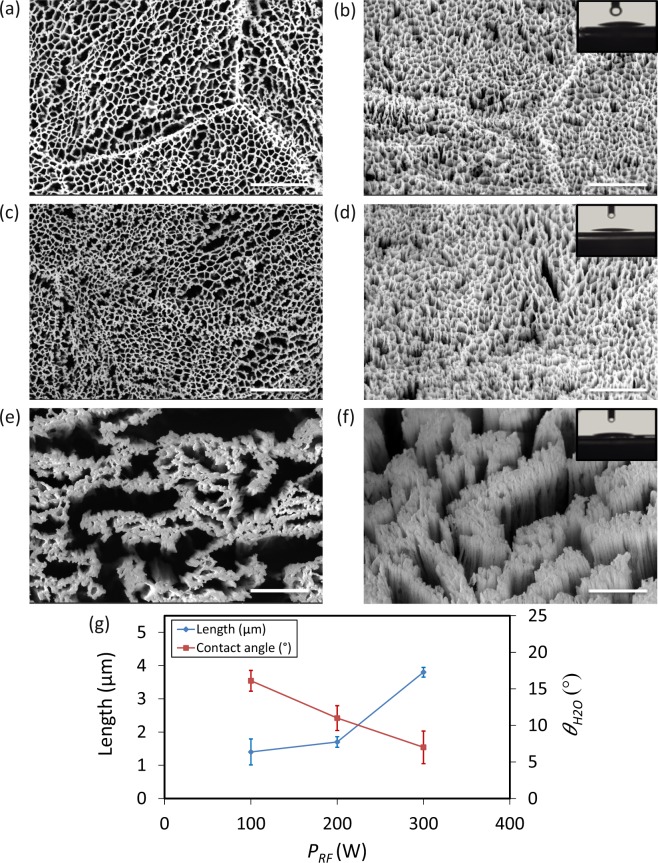
SEM micrographs of the Ti nanostructures created using different values of RF power: (**a,b**) 100 W, (**c,d**) 200 W, (**e,f**) 300 W. The left sub-figures were taken from the top view while the right sub-figures were taken at 35° tilted view. Other etching conditions: Δ*t* 20 min, Cl_2_ 30 sccm, Ar 2.5 sccm, *T* 20 °C, *p* 0.02 mbar and *P*_*ICP*_ 600 W. Insets show a droplet of water on the black Ti surface. The scale bar is 1 µm. (**g**) The length and wettability of the Ti nanostructures as a function of *P*_*RF*_.

When *P*_*RF*_ varied between 100 W and 200 W, the nanostructures were connected to each other and formed a network (Fig. 3a,c). There were no significant differences between the lengths of the nanostructures (Fig. 3b,d,g, and Table S1). Increasing *P*_*RF*_ to 300 W resulted in a large increase in the length of nanostructures from 1.4 µm to 3.8 µm (Fig. 3f,g, and Table S1). Furthermore, the power was enough to break the struts and separate the nanostructures from each other creating nanopillar clusters instead of an overall network (Fig. 3e). Moreover, the average spacing between nanopillars significantly increased (Fig. 3e,f). All structures created under these conditions were highly hydrophilic (insets of Fig. 3b,d,f, as well as Fig. 3g).

### The effects of ICP source power

**Figure 4:**
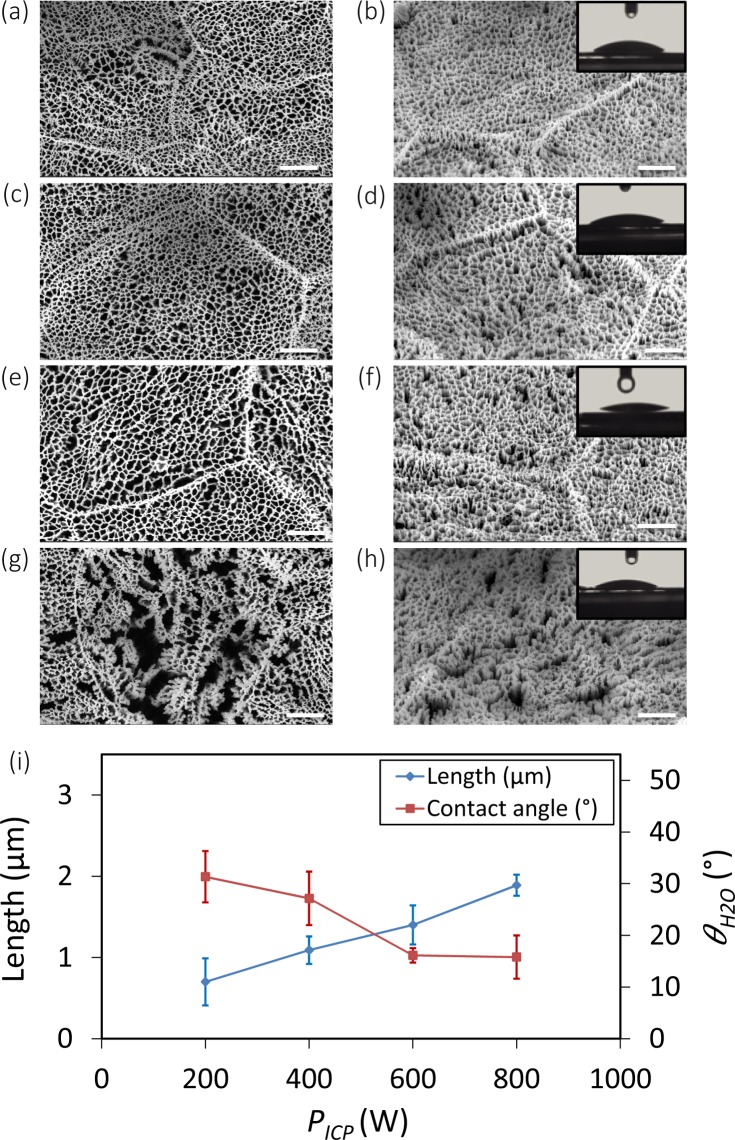
SEM micrographs of the Ti nanostructures created using different values of *P*_*ICP*_: (**a,b**) 200 W, (**c,d**) 400 W, (**e,f**) 600 W, and (**g,h**) 800 W. The left sub-figures were taken from the top view while the right sub-figures were taken at 35° tilted view. Other etching conditions: Δ*t* 20 min, Cl_2_ 30 sccm, Ar 2.5 sccm, *T* 20 °C, *p* 0.02 mbar, and *P*_*RF*_ 100 W. Insets show a droplet of water on the black Ti surface. The scale bar is 1 µm. (i) The length and wettability of the Ti nanostructures as a function of *P*_*ICP*_.

There was an approximately linear relationship between increasing *P*_*ICP*_ and the length of the nanostructures (Fig. 4i). Increasing *P*_*ICP*_ from 200 W to 800 W also had an effect on the pore size of the networked structure (Fig. 4a,c,e,g). At 200 W, the surface was covered with short nanopillars, which were densely connected to each other in a fine network (Fig. 4a). At 400 W and 600 W, nanostructures started to separate from each other, which made the pore size larger (Fig. 4c,e). At 800 W, *P*_*ICP*_ was enough to break the struts and create clusters of nanopillars instead of a highly networked nanostructure (Fig. 4g). Although the surfaces obtained at 200 and 400 W were hydrophilic (insets of Fig. 4b,d,i), increasing *P*_*ICP*_ to 600 W and 800 W made the surfaces superhydrophilic (insets of Fig. 4f,h, as well as i).

### The effects of chamber pressure

**Figure 5:**
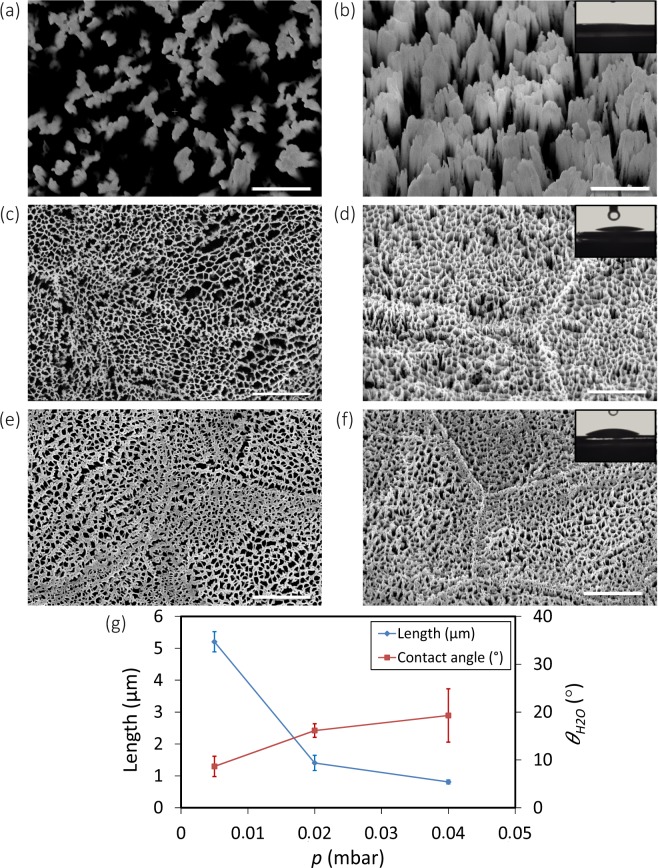
SEM micrographs of the Ti nanostructures created using different values of the chamber pressure: (**a,b**) 0.005 mbar, (**c,d**) 0.02 mbar, and (**e,f**) 0.04 mbar. The left sub-figures were taken from the top view while the right sub-figures were taken at 35° tilted view. Other etching conditions: Δ*t* 20 min, Cl_2_ 30 sccm, Ar 2.5 sccm, and *T* 20 °C. *P*_*ICP*_ and *P*_*RF*_ were 600 W and 100 W, respectively. Insets show a droplet of water on the black Ti surface. The scale bar is 1 µm. (**g**) The length and wettability of the Ti nanostructures as a function of *p*.

The effects of chamber pressure showed an opposite effect on morphology and wettability of the etched surfaces. As the pressure increased, the length and wettability of the nanopillars decreased. In addition, the etching was more omnidirectional and pillar sidewalls were not vertical anymore (Fig. 5f). Moreover, at low pressures, there was almost no inter-pillar connection between the nanopillars (Fig. 5a,b).

For the structures created at 0.005 mbar, water droplet immediately disappeared and sank between pillars (inset of Fig. 5b), and the surface was superhydrophilic (inset of Fig. 5b,g). At higher pressures (*e.g*., 0.02 mbar and 0.04 mbar), although the surface was hydrophilic, the contact angle was higher (insets of Fig. 5d,f,g).

### The effects of temperature

**Figure 6:**
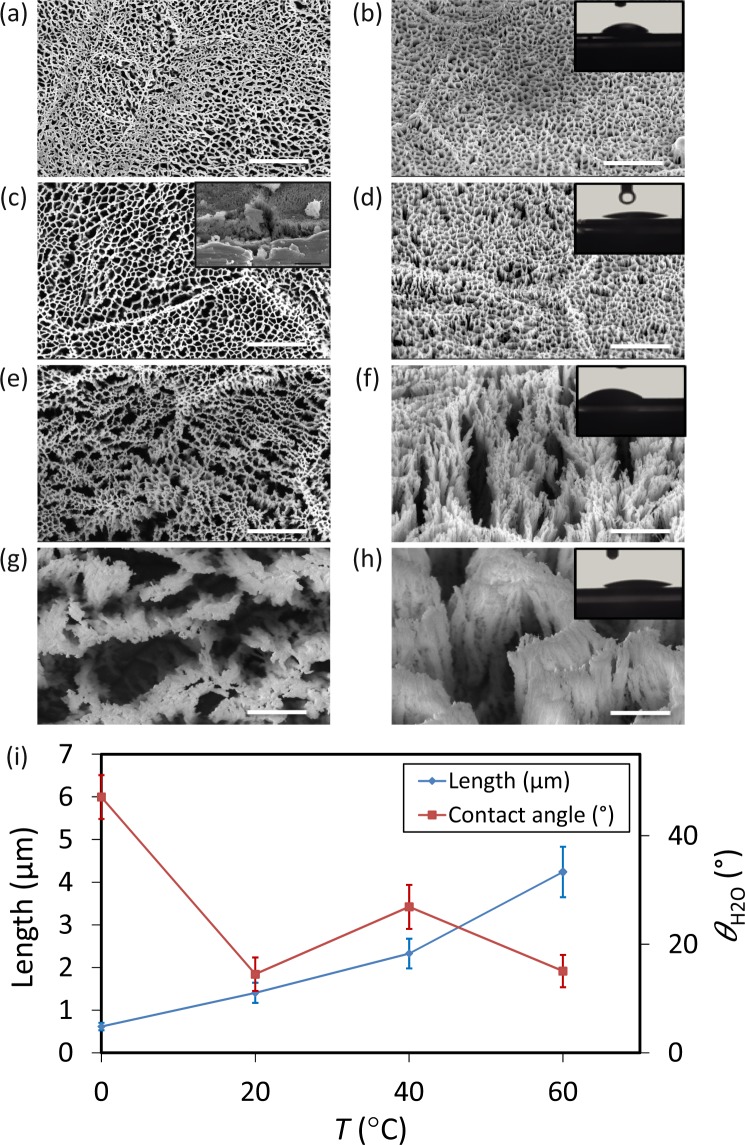
SEM micrographs of the Ti nanostructures created using different values of the chamber temperature: (**a,b**) 0 °C, (**c,d**) 20 °C, (**e,f**) 40 °C and (**g,h**) 60 °C. The left sub-figures were taken from the top view while the right sub-figures were taken at 35° tilted view. The inset in figure (**c**) is a representative cross-section image used to measure the length of the nanostructures (the scale bar is 1 µm). Other etching condition: Δ*t* 20 min, Cl_2_ 30 sccm, Ar 2.5 sccm, and *p* 0.02 mbar. *P*_*ICP*_ and *P*_*RF*_ were 600 W and 100 W, respectively. Insets in b-d-f-h show a droplet of water on the black Ti surface. The scale bar is 1 µm. (**i**) The length and wettability of the Ti nanostructures as a function of *T*.

For the sample produced at 0 °C, the surface was relatively flat, nanostructures were densely connected to each other (Fig. 6a,b). The length of the nanopillars was 0.6 ± 0.1 µm under this condition (Fig. 6i, and Table S1).

As the temperature increased to 20 °C, the etching rate of sidewalls was enhanced, and nanostructures started to separate from each other (Fig. 6c,d). At higher temperatures (*e.g*., 40 °C and 60 °C), taller nanopillars and larger pores were formed (Fig. 6e,g). Individual nanopillars were recognizable and made it possible to measure their diameter (Fig. 6f,h). Independent of the shape and profile of the nanostructures, the etching rate was almost linear from 0 °C to 40 °C (Fig. 6i). At 60 °C, a sharp increment was observed (Fig. 6g,i, and Table S1).

Surface wettability measurement indicated that for 0 °C, the water contact angle was 47.1 ± 4.0° (inset of Fig. 6b,i). For 20 °C, the surface was hydrophilic with a contact angle of 16.1 ± 1.4° (Fig. 6d,i). At 60 °C, the contact angle was almost the same as for 20 °C (Fig. 6h,i).

### The effects of gas composition

#### Chlorine gas flow rate

**Figure 7:**
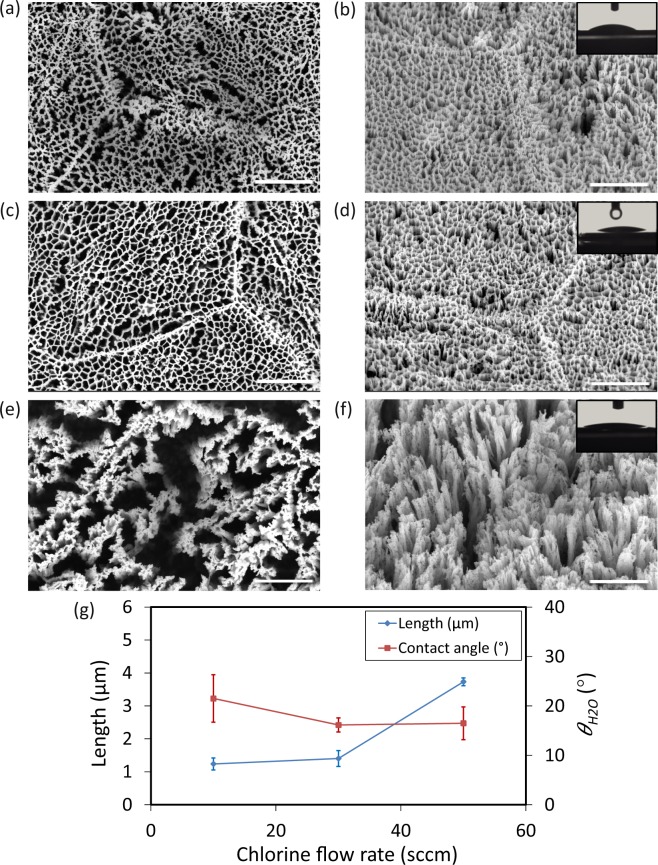
SEM micrographs of the Ti nanostructures created using different values of the Cl_2_ pressure: (**a,b**) 10 sccm, (**c,d**) 30 sccm, and (**e,f**) 50 sccm. The left sub-figures were taken from the top view while the right sub-figures were taken at 35° tilted view. Other etching conditions: Δ*t* 20 min, *p* 0.02 mbar, Ar 2.5 sccm, and *T* 20 °C. *P*_*ICP*_ and *P*_*RF*_ were 600 W and 100 W, respectively. Insets show a droplet of water on the black Ti surface. The scale bar is 1 µm. (**g**) The length and wettability of the Ti nanostructures as a function of the Cl_2_ flow rate.

The length of the nanopillars slightly increased from 1.2 ± 0.2 µm to 1.4 ± 0.2 µm when the Cl_2_ flow rate increased from 10 sccm to 30 sccm (Fig. 7b,d,g, and Table S1). Increasing Cl_2_ flow rate from 30 sccm to 50 sccm led to a large increment in the length of the nanostructures (Fig. 7d,f,g, and Table S1). Similar to the case of higher temperatures, the nanostructures separated from each other and clusters formed (compare Fig. 7c with e). In this case, one can define nanopillar diameter and measure it (Fig. 7f). The average length for nanopillars was 3.7 ± 0.1 µm and the diameter was 31.9 ± 9.3 nm (Fig. 7g and Table S1). The surface was more hydrophilic when it was decorated with lengthy nanopillars with larger interspaces (insets of Fig. 7b,d,f,g).

#### Argon gas flow rate

**Figure 8:**
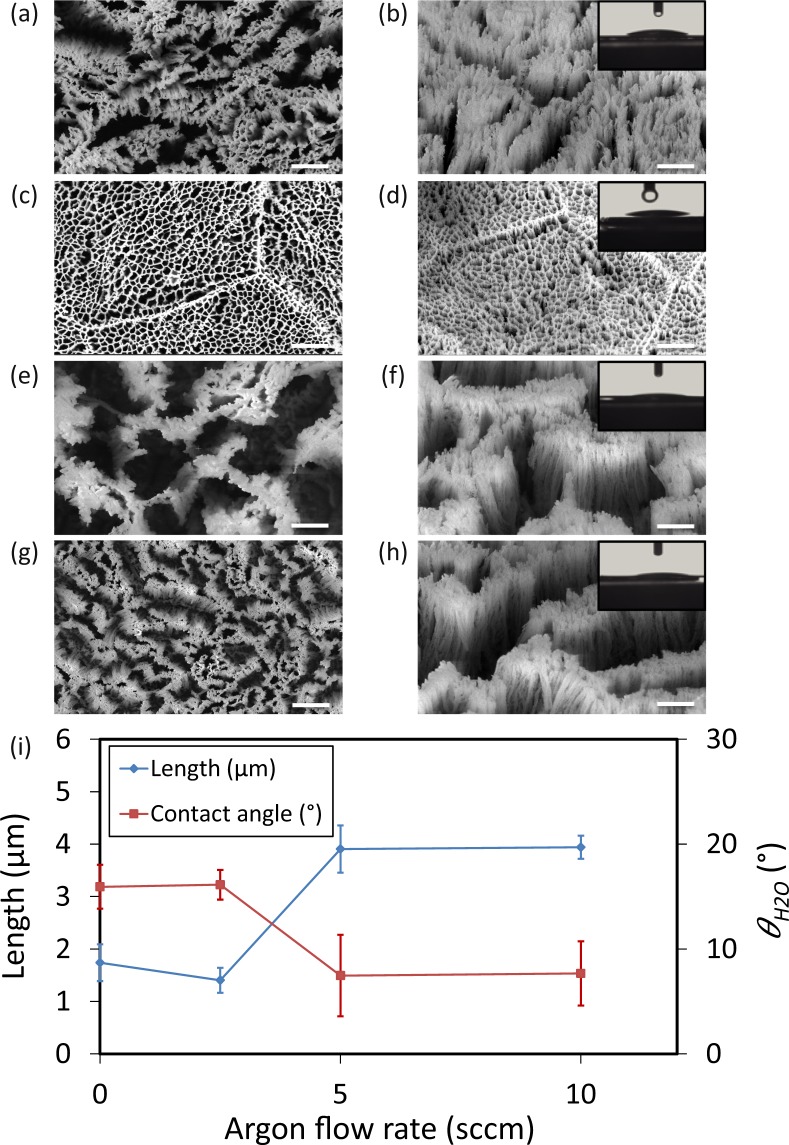
SEM micrographs of the Ti nanostructures created using different values of the Ar pressure: (**a,b**) 0 sccm, (**c,d**) 2.5 sccm, (**e,f**) 5 sccm, and (**g,h**) 10 sccm. The left sub-figures were taken from the top view while the right sub-figures were taken at 35° tilted view. Other etching conditions: Δ*t* 20 min, *p* 0.02 mbar, Cl_2_ 30 sccm, and *T* 20 °C. *P*_*ICP*_ and *P*_*RF*_ were 600 W and 100 W, respectively. Insets show a droplet of water on the black Ti surface. The scale bar is 1 µm. (**i**) The length and wettability of the Ti nanostructures as a function of the Ar flow rate.

The average length of the nanostructures without using Ar was 1.7 ± 0.3 µm and the diameter for a single nanopillar was 27.8 ± 8.6 nm (Fig. 8b,i, and Table S1). When Ar gas was introduced into the chamber, the length of nanopillars slightly decreased, and they created a network instead of clusters of nanopillars (Fig. 8c,d). However, by increasing Ar flow rate from 2.5 sccm to 5 sccm, the length started to increase from 1.4 µm to 3.9 µm (Fig. 8f,i, and Table S1). As Ar flow was further increased to 10 sccm, the length remained relatively constant (Fig. 8i). As the Ar flow rate increased, nanopillars were not vertical anymore and tended to bend in one direction (Fig. 8g). In other words, increased Ar flow rates caused isotropic etching and created a roughness on nanopillar sidewalls (Fig. 8e–h). Moreover, at Ar flow rates of 5 and 10 sccm, nanopillars were densely connected to each other and the tip was not sharp anymore (Fig. 8f,h).

**Figure 9:**
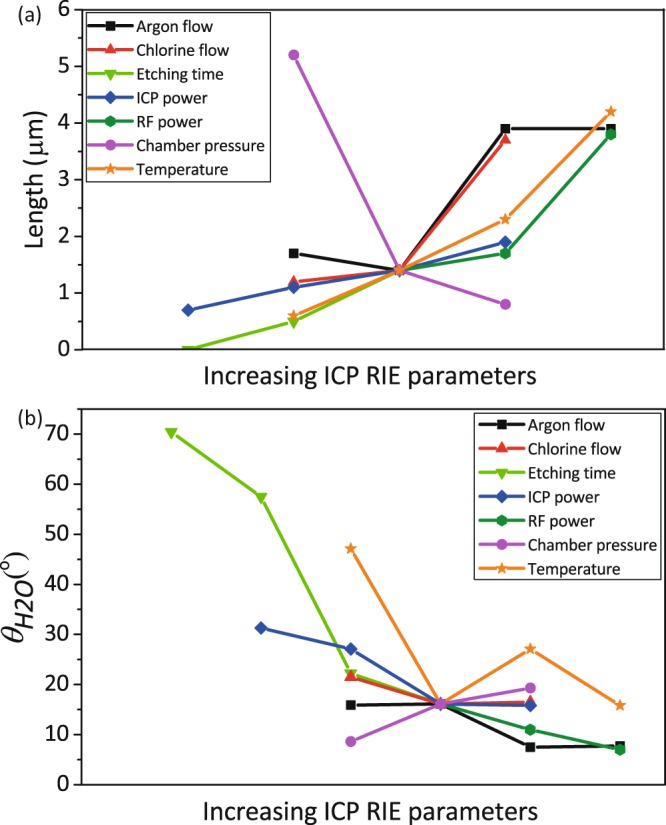
The change in the length (**a**), and wettability (**b**) of the Ti nanostructures when the processing parameters of ICP RIE are varied.

Contact angle measurement indicated that black Ti nanostructures created at Ar flow rates of 5 and 10 sccm were superhydrophilic, with contact angles of 7.5° ± 3.9° and 7.7° ± 3.1°, respectively (insets of Fig. 8f,h,i). However, for lower Ar flow rates, the contact angle was 15.9° ± 2.1° and 16.1° ± 1.4° for 0 and 2.5 sccm, respectively (insets of Fig. 8b,d, and Fig. 8i). A comprehensive overview of the length and contact angle of the created nanostructures as functions of processing parameters is presented in Fig. 9.

### Mechanical tests

**Figure 10:**
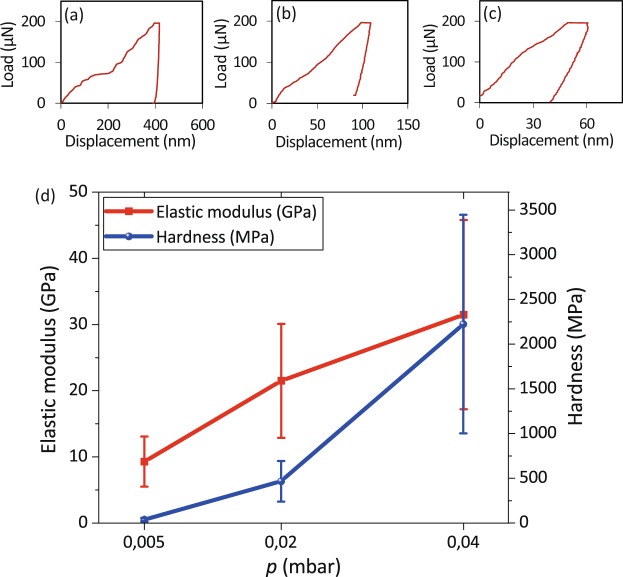
The load-displacement curves for the Ti nanostructures created using chamber pressures of (**a**) 0.005, (**b**) 0.02, and (**c**) 0.04 mbar. (**d**) The elastic modulus and hardness of the black Ti nanostructures created at different chamber pressures.

Black Ti produced at the higher chamber pressure (0.04 mbar) exhibited the highest values of the relative elastic modulus (31.5 ± 14.3 GPa) and relative hardness (2224 ± 1220 MPa) as well as the lowest values of the indentation depth (Fig. 10c,d). Decreasing the chamber pressure to 0.02 mbar resulted in an elastic modulus of 19.4 ± 8.2 GPa and a hardness of 440.6 ± 231.7 MPa (Fig. 10b,d). At 0.005 mbar, the elastic modulus and hardness (9.2 ± 0.9 GPa and 47 ± 4.2 MPa, respectively) were much lower (Fig. 10a,d).

### Biofunctionality of black Ti surfaces

**Figure 11:**
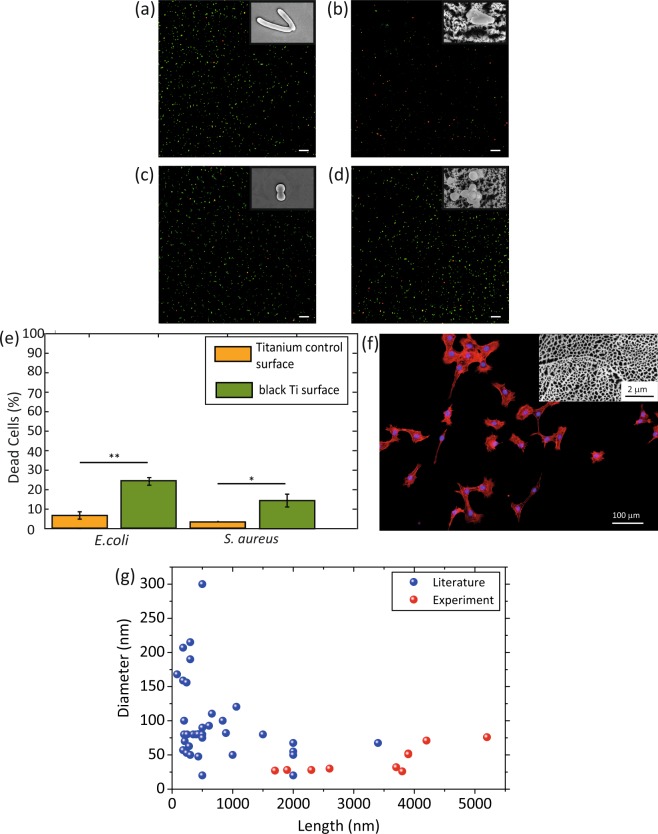
(**a,b**) The live/dead images of *E.coli* and (**c,d**) *S. aureus* cultured for 18 hr on the control (left) and black Ti (right) surfaces. Insets show the bacterial cell morphology on the determined surface. The black Ti surfaces were created using the following parameters: *P*_*ICP*_ = 400 W, *P*_*RF*_ = 100 W, Δ*t* = 20 min, *p* = 0.02 mbar, the flow rate of Cl_2_ = 30 sccm, and the flow rate of Ar = 2.5 sccm. The scale bar is 500 µm. (**e**) Quantitative characterization of the percentage of damaged *E. coli* and *S. aureus* bacterial cells on black Ti and titanium control surfaces (*p < 0.05 and **p < 0.01). (**f**) Fluorescence microscope image of actin cytoskeleton (red) and nucleus (blue) of preosteoblast cells attached to the black Ti surface (produced in the same ICP RIE condition) after 1 day of culture. Inset shows the black Ti morphology. (**g**) Comparison between the dimensions of bactericidal nanopatterns reported in the literature (blue bullets) and the dimensions of the black Ti nanopillars created in the current study (red bullets), indicates that the length and diameter of our black Ti nanopillars are within the range of the bactericidal nanopatterns reported in the literature.

Preliminary biological tests were performed with the selected ICP RIE titanium surface. The results of bacterial culture showed that this ICP RIE structure could change the morphology of Gram-negative and Gram-positive bacteria (Fig. 11a–d). By comparison, no significant deformation in shape was found for *E. coli* and *S. aureus* on the titanium control surface (Fig. 11a,c). On the ICP RIE surface, the nanopillars were not deformed by the bacteria cells and the surface showed a significantly higher bactericidal efficiency against *S. aureus*, (*i.e*. 14.6 ± 6.6%) as compared to the smooth titanium surface (*i.e*. 3.2 ± 0.2%) (Fig. 11e). Moreover, while the bactericidal efficiency of the control surface for *E. coli* was 6.5 ± 3.8%, (Fig. 11a), the black Ti surface showed a bactericidal efficiency of 24.4 ± 3.9% (Fig. 11b,e). Although low bactericidal efficiencies were obtained with this specific surface, the findings indicate the bactericidal potential of such surfaces. In addition, the same surface showed no adverse effects on the attachment and spreading of preosteoblast cells, as indicated by staining cell nucleus and actin filaments (Fig. 11f).

## Discussion

ICP RIE chlorine-based titanium etching is based on chemical isotropic and physical anisotropic etching^43^. Considering that the etching mechanism to create black Ti is the same as the process that is reported in the literature for thin film titanium, TiCl_4_ and TiCl_2_ are the reaction products^43^. The main component of nanostructures after ICP process was TiO_x_ (Fig. 2h). The origin of formation of the ICP RIE nanostructures is the existence of etch inhibitors. Etch inhibitors are generated by sputtering the Al-containing particles from the chamber sidewalls or the carrier wafer. Oxygen from the sapphire carrier wafer act as local etch-inhibitors and form local nanomasks of TiO_x_ on the etched surface. Moreover, Ti easily becomes oxidised by being exposed in air. Therefore, the existence of O in EDX characterization is because of TiO_x_ nanomasking and native oxide layer. The reaction products and radicals inside the plasma, also could easily stick to the substrate and act as nanomask for the substrate underneath^45^. These nanomasks block the surface underneath against the anisotropic ion bombardment. Since the ions etch the substrate uniformly, the existence of these local nanomasks leads to different etching rate of the surface and creation of nanostructures.

### Etching time

After 1 min etching, the contact angle of the resultant profile was the same as the control titanium surface (Fig. 2g). This can be explained by the fact that nanostructures had not formed yet. When the etching time increased to 5 min, nanostructures appeared on the surface (Fig. 2c,d). The shape of the nanostructures after 20 min etching was the same as 5 min, but they were larger in length (Fig. 2f,g). Increasing etching time made the surface more hydrophilic (Fig. 2g). Length and interspace could play an important role in the surface wettability. The findings indicated that longer nanostructures were more hydrophilic. This trend was the same for all of the nanostructures created under different conditions. Nevertheless, determining the interspace was not feasible in these types of randomly oriented nanostructures.

### RF power

In ICP RIE, *P*_*RF*_ is used to control ion energy, whereas *P*_*ICP*_ controls the ion flux. More details and the relationships between them can be found in the supplementary document. By increasing *P*_*RF*_, ions with higher energy and acceleration, physically bombard the substrate and make the etching process faster and more directional. Increasing *P*_*RF*_ from 100 W to 200 W, slightly increased the etching rate. There was no significant difference in the shape of the resultant profile. However, the length changed from 1.4 ± 0.2 µm to 1.7 ± 0.1 µm (Fig. 3b,d,g). With increasing *P*_*RF*_ to 300 W, the etching rate substantially increased, and the length of the profile reached 3.8 ± 0.4 µm (Fig. 3f,g). As previously mentioned, titanium etching mostly relies on the chemical process^43^. For low *P*_*RF*_ (*i.e*. 100 W and 200 W), the ion energy probably was not high enough to change the working regime, and the etching rate was dependent on other parameters controlling the chemical process. While for 300 W, ion energy was enough to remove the material from the substrate, and the dominant process was physical directional etching. Therefore, the final profile was anisotropic, nanopillars were separated from each other, and nanopillar sidewalls became vertical.

### ICP power

The main function of *P*_*ICP*_ is creating a DC bias voltage to accelerate ion flux towards the cathode, where the wafer is placed. Increasing *P*_*ICP*_ leads to dissociation and ionization of Cl_2_ molecules into Cl atoms, thereby speeding up this process and increasing ions, radicals, and reactive species. The etching rate of titanium was almost a linear function of *P*_*ICP*_ (Fig. 4i). For *P*_*ICP*_ below 800 W, the chemical process was dominant, the etching was anisotropic, and nanostructures were connected to each other (Fig. 4a–f). For a *P*_*ICP*_ of 800 W, it seems that a combination of chemical and physical processes was involved. Therefore, the resultant profile was more directional (Fig. 4g,h).

### Chamber pressure

The effects of chamber pressure on the characteristics of the nanostructures were also studied. Based on Eq. (1), the mean free path for Cl etching atoms can be obtained as:

1 $$\lambda =\frac{kT}{\sqrt{2}\pi {\sigma }^{2}p}$$

where *λ* is the mean free path, *k* is the Boltzmann constant, *T* is the temperature, *σ* is the particle diameter, and *p* is the chamber pressure. *p* has a reverse relationship with the mean free path^46^. This means that at lower pressures, the collision between particles is decreased and the etching process is faster and more directional. In this regime, physical etching and ion bombardment play more important roles in etching. Increasing chamber pressure increases the randomized collision between particles, reduces the energy of radicals and ions, and leads to a decrease in the etching rate and non-vertical sidewalls for the final nanostructures. At a chamber pressure of 0.005 mbar, the nanopillar length was 5.2 ± 0.3 µm. Increasing the chamber pressure to 0.02 mbar made the etching process much slower with a nanostructure length of 1.4 ± 0.2 µm. Continuing to increase the chamber pressure setting it to 0.04 mbar changed the length of the nanostructures to 0.8 ± 0.1 µm. The dominancy of the etching process had an effect on the roughness of the sidewalls. For instance, the black Ti nanostructures produced at 0.005 mbar chamber pressure had smoother sidewalls as compared with the samples produced at 0.04 mbar (Fig. 5b,f). Furthermore, there were no struts between the nanopillars produced at low pressures (Fig. 5a).

### Temperature

The chamber temperature is one of the parameters that has an impact on the morphology of the resulting profile. At low temperatures, the etching rate of the sidewalls is negligible^47^. At 0 °C, the surfaces were mostly flat (Fig. 6a), due to the low etching rates of the sidewalls. Increasing the temperature to 20 °C resulted in a higher etching rate of the sidewalls (Fig. 6d,i). As the reaction probability of Cl radicals with the substrate depends on the substrate temperature, at higher temperatures, Cl radicals react with sidewalls and make the etching less directional and more isotropic. The overall Ti etching rate is increased at higher temperatures (Fig. 6e–h).

### Etching gases

The combination of Cl_2_ and Ar gasses is one of the suitable mixtures to create black Ti. Although SF_6_ can be also used as the etching gas, most of the published reports have used Cl_2_ and Ar as the etchant gases^40,43,48,49^. This is because the etching profile created using SF_6_ is isotropic, making it unsuitable for solar and microelectronic applications (*i.e*. the most common areas of applications for such profiles)^50^. Furthermore, the resulting nanostructures of the Cl_2_ gas have vertical sidewalls and smoother surfaces^51^. We therefore chose Cl_2_/Ar chemistry and studied the effects of gas flows on the Ti structures.

### Chlorine gas flow rate

As previously discussed, in Cl_2_-based dry etching process of titanium, the reaction products are TiCl_4_ and TiCl_2_^43^. During this process, the Cl_2_ molecules break down, ionize, and turn into atomic Cl or Cl ions. By increasing the rate of introducing Cl_2_ molecules into the chamber, the ionization and dissociation rate increase, and more active species are available to increase the etching rate. For low flow rates of Cl_2_, the limiting factor is the amount of Cl_2_ gas present inside the chamber. The Cl_2_ can therefore not flow through the network, reach the surface, and increase the etching rate. Consequently, no significant increases were observed when the Cl_2_ flow rate was increased from 10 sccm to 30 sccm (Fig. 7g). At higher Cl_2_ flow rates, the amount of Cl_2_ seems to be adequate to break the struts and increase the etching rate (Fig. 7e,g).

### Argon gas flow rate

Ar is one of the gasses that is widely used to etch titanium due to its high selectivity against TiO_2_, SU8, and Ni that are suitable masks to etch titanium for solar cells and MEMS applications^50^. Furthermore, some studies have shown that the existence of inert gasses during the etching process leads to process uniformity and improves plasma stability without changing the chamber pressure^43,52^. Efremov *et al*. have reported that at a constant pressure, introducing Ar to a Chlorine plasma leads to increased etching rate for different types of materials^53^. In this case, additional atoms leave the surface and the quality of plasma is increased, resulting in a higher etching rate^52^. Without introducing Ar into the chamber, the length of the nanopillars was 1.7 ± 0.3 µm (Fig. 8i). With introducing Ar into the chamber, *i.e*., 2.5 sccm, although the bias voltage is increased, the density of chlorine etching species is decreased, resulting in a lower total etching rate^54^ (length = 1.4 ± 0.2 µm) (Fig. 8c,i). At higher Ar flow rates, *i.e*., 5 sccm, the DC bias voltage becomes higher and ion flux bombards the substrate more severely, leading to higher etching rate with more anisotropicity. A sharp increase was also observed in the length of the nanopillars to 3.9 ± 0.4 µm. One possible scenario for this behavior is increasing bombardment of the substrate by high energy active species and enhancement of ion enhanced etching^43^. Increasing Ar flow rate to 10 sccm did not change the resultant profile (Fig. 8f,h). The reason can be explained by the fact that although ion bombardment is intense in this situation, the amount of available chlorine species is not enough to increase the etching rate^54^. On the other hand, increasing Ar flow rate makes nanopillars bended. The possible mechanism for bending of the nanopillars is that increasing Ar flow rate into the chamber lead to increased surface roughness^43^. Roughness on the edge of TiO_x_ nanomasks leads to mask underetching. As in the case of thin films etching, mask underetching and rough sidewalls of the created nanopillars leads to non-vertical/tilted sidewalls, and therefore bended nanopillars.

The influence of different ICP RIE parameters on the length of the nanopillars and the contact angles are summarized in Fig. 9. Using both graphs presented in this figure, one can determine which parameter has a major impact on the nanopillar length and surface wettability. For instance, reducing the chamber pressure to 0.005 gives the possibility to produce superhyrophilic high aspect ratio black Ti nanopillars. Another important aspect that can be understood from this figure is the trend of change in the nanopillar length and contact angle as a result of varying different processing parameters. For instance, the slope of the curve for *T* and *P*_*ICP*_ is approximately linear. These linear curves could then be used to estimate the required value of a specific parameter to achieve the desired dimensions.

We studied the effects of the chamber pressure on the mechanical response of black Ti using nanoindentation tests. The elastic modulus, hardness, and indentation depth were extracted from the force-displacement curves (Fig. 10a–c). Based on Eq. (2)^55^:

2 $$H{\boldsymbol{=}}\frac{{P}_{{\max }}}{{A}_{c}}\,$$

hardness (*H*) can be calculated by dividing the maximum applied load (*P*_*max*_) by the contact area at the peak load (*A*_*C*_). The indentation modulus (E_r_) can be determined using the initial slope of the unloading curves:

3 $$S=\frac{dP}{dh}=\frac{2}{\sqrt{\pi }}{E}_{r}\sqrt{{A}_{C}}$$

where S in the slope of the upper part of the unloading curve during initial stages of unloading, P is the load, and h is the displacement relative to the initial undeformed surface. The Eq. (4) shows the relationship between the mechanical properties of the specimen and the nanoindenter tip^22,55^:

4 $$\frac{1}{{E}_{r}}=\frac{(1-{\nu }_{m}^{2})}{{E}_{m}}+\frac{(1-{\nu }_{i}^{2})}{{E}_{i}}$$

where *v*_*m*_ and *E*_*m*_ are the Poisson’s ratio and the elastic modulus of the specimen, *v*_*i*_ and *E*_*i*_ are the Poisson’s ratio and elastic modulus of a nanoindenter tip, and *E*_*r*_ is the indentation modulus. The Poisson’s ratio of black Ti was assumed to be the same as bulk TiO_2_, which is 0.28^22,55^.

It is reported in the literature that the elastic modulus of TiO_2_-based nanostructures is between 4 and 43 GPa, depending on the applied load, fabrication method, and the crystalline orientation of the nanostructures^22,56^. The results indicated that black Ti produced at higher chamber pressures (*i.e*. 0.04 mbar) were more robust and had higher overall elastic modulus and hardness (Fig. 10d). That is likely due to the presence of highly dense, flat, and connected nanostructures (Fig. 5e), which make the surface behave almost the same as the bulk material with low percentages of porosity and high levels of resistance against the penetration of the tip (Fig. 10c). As mentioned before, at lower pressures (*e.g*., 0.005 mbar), black Ti nanopillars were longer in length and separated from each other (Fig. 5a,b). These two characteristics made them less robust against the penetration of the nanoindenter tip. At small displacements for black Ti nanostructures, the deformation was elastic. As the applied force and penetration depth increased, the nanopillars started to bend and crush. Therefore, the contact area was compact, which led to the densification of the nanostructures. The densification increased with the applied force and made the structure less porous and more robust. The maximum obtainable value of the elastic modulus in this case happened when the structure was fully dense (the same as bulk material)^55^.

It is known that surfaces covered by micro- and nano-topographical features exhibit bactericidal properties within a distinct range of dimensions of nanotopographies including height (length), diameter, and interspacing^4,5,9,28^. Nanopillars with a length between 180 nm and 3.4 µm and diameters in the range of 20–300 nm are known to be bactericidal^57^. Most of the nanostructures achieved in this study were within those two ranges and are therefore expected to be bactericidal (Fig. 11f). For some of the highly connected nanostructures, it was not feasible to determine the diameter. Thus, they are not plotted in Fig. 11f.

The bactericidal activity of the specific black Ti surface tested preliminarily against *E. coli* was comparable to previously studied naturally occurring nanopatterns of gecko skin and cicada wing^39,58,59^. However, a higher bactericidal efficiency of black Ti against *S. aureus* (≈76%) has been reported by Hasan *et al*. after 24 hours of incubation^40^. Nevertheless, the surfaces are not similar and, in our study, we did not optimize the structures for achieving the maximum bactericidal activity. In addition, the cytocompatibility test of the same black Ti surface (Fig. 11f) performed with preosteoblast cells indicated differential effects towards different types of living organisms which makes such a surface a proper candidate for biofunctionalization of bone implants.

The commonly accepted theory addressing the bactericidal behavior of nanopatterned surfaces is the direct penetration of high aspect ratio nanostructures into the bacterial cell wall and killing it via mechanical rupturing of the cell wall^60^. However, many other factors such as the affinity of the bacterial cell to the surface, the cell wall rigidity, and the contact area between the bacterial cell and the nanopatterns could also affect the bactericidal efficiency^4,40,58,61^. For instance, Hasan *et al*.^40^ and Linklater *et al*.^61^, have reached higher bactericidal efficiency with RIE Ti nanostructures by keeping the chamber pressure at lower values (*i.e*., 3 mTorr ~0.004 mbar) which leads to more separated nanopillars. Etching time was an important factor which made a huge difference between the bactericidal efficiency in their experiment. However, the optimum ICP RIE structure for a maximum bactericidal activity is yet to be established for both Gram-negative and Gram-positive bacteria.

## Methods

### Titanium specimens

Annealed titanium foils with a thickness of 125 µm were used for this study (99.96% purity, Goodfellow, Huntingdon, UK). The foils were cut to the size of a 4-inch (diameter = 10.16 cm) silicon wafer and polished by chemical-mechanical polishing (CMP Mecapol E460, Saint-Martin-le-Vinoux, France). After polishing, the whole surface was covered with photoresist to protect the sample against possible damages. Using a Disco dicer (Disco Hi-Tec Europe GMbH, Munich, Germany), the 4-inch titanium foil was cut into 8 × 8 mm^2^ pieces. After cutting, the photoresist layer was removed by acetone and the samples were cleaned in ethanol and isopropyl alcohol (IPA), respectively, and then spin-dried. The polished titanium samples were used for ICP RIE experiments.

### Roughness measurements

The surface roughness after polishing was measured by atomic force microscopy (AFM) (Bruker Dimension FastScan, Santa Barbara, USA) in the ScanAsyst mode with a FastScan-A tip (the same manufacturer) with a nominal spring constant of 17 N/m and a nominal resonance frequency of 1400 kHz. Three different specimens were analyzed by performing measurements on three different areas of 30 × 30 µm^2^ for each specimen. The roughness parameters *R*_*a*_ (arithmetic roughness average) and *R*_*q*_ (root mean square roughness) were determined for the polished specimens. The reported values represent the mean and standard deviation (SD).

### Surface chemical composition

The chemical composition of the specimens before and after ICP RIE process was characterized using an energy-dispersive X-ray spectroscopy (EDX) analysis performed inside an scanning electron microscope (SEM) (FEI NovaNano SEM 450, Hillsboro, USA) using images with × 13000 magnification acquired with an accelerating voltage of 10 kV.

### Surface modification by ICP RIE

The experiments were performed using an ICP RIE machine (PlasmaLab System 100, Oxford Instruments, UK). The etching process was performed with Cl_2_ and Ar gases, and the pressure of He used as the back-cooling gas was kept at 8 Torr. The polished specimens were glued with diffusion oil on a 4-inch sapphire wafer as a carrier. After completion of the etching process, the samples were cleaned in ethanol for 15 min, acetone for 15 min, and IPA. The effects of seven different ICP RIE processing parameters were systematically investigated (Table 1). The starting conditions were as follows: ICP source power = 600 W, RF power = 100 W, temperature = 20 °C, chamber pressure = 0.02 mbar, etching time = 20 min, Cl_2_ flow rate = 30 sccm, and Ar flow rate = 2.5 sccm. In every experiment, one single processing parameter was varied while keeping the other parameters constant in order to delineate the effects of each individual parameter.

**Table 1:** The processing parameters of ICP RIE considered in this study. The values typed in bold define the starting (*i.e*. reference) set of conditions.

Process variable (unit)	Abbreviation	Values
ICP Power (W)	*P*_*ICP*_	200–400–**600**–800
RF Power (W)	*P*_*RF*_	**100**–200–300
Etching time (min)	Δ*t*	0–1–5–**20**
Pressure (mbar)	*P*	0.005–**0.02**–0.04
Temperature (°C)	*T*	0–**20**–40–60
Cl_2_ flow (sccm)	—	10–**30**–50
Ar flow (sccm)	—	0–**2.5**–5–10

### Scanning electron microscopy imaging of the modified surfaces

High-resolution images of the etched surfaces were obtained by field emission SEM (Helios NanoLab 600i dualbeam, FEI, Hillsboro, USA) at 5 kV and 86 pA. Top views and tilted images (35°) were acquired at increasing magnifications (×2,000, ×5,000, ×10,000, ×20,000, ×40,000, and ×80,000). For each angle, three different regions on three different specimens were characterized to assess the uniformity and reproducibility of the etched topographies.

A scratch was made on the surface of the samples using a tweezer. Then, SEM images were taken at 35° to measure the length of the nanostructures across the scratched line (inset of Fig. 6c).

### Contact angle measurements

The static contact angle of the surfaces before and after the ICP RIE experiments was measured by a drop shape analyzer (DSA 100, Kruss, Hamburg, Germany) using deionized water. A volume of 1 μl liquid with a falling rate of 60 µl/min was placed on the surface using a syringe. All of the contact angle measurements were carried out two days after plasma processing and the contact angle figures were recorded 5 s after the droplet had rested on the surface. The reported value for each measurement is the average of three different measurements on three different specimens.

### Nanoindentation experiments

Nanoindentation experiments were performed to determine the hardness and elastic modulus of the etched titanium specimens. The experiments were carried out under load control model, with the loading speed of 10 µN/s and the loading time of 10 s, using a Nano Indenter G200 (KLA-Tencor, California, USA) on black Ti specimens produced at different chamber pressures of 0.005, 0.02, and 0.04 mbar and under two different maximum applied loads of 200 µN and 2 mN. A diamond Berkovich tip (elastic modulus = 1140 GPa, Poisson’s ratio = 0.07) was used to conduct the experiments. Under a load of 2 mN, the penetration depth was around 600 nm, which was around the length of the nanostructures produced at 0.04 mbar, meaning that the titanium substrate could affect the measurements. The maximum applied load was therefore chosen as 200 µN in all experiments based on the literature and on the above-mentioned experimental results. For each specimen, a series of 5 × 5 indents were performed on a tightly spaced grid, with spacing of 10 µm between each indent. The reported values are therefore the average and standard deviation of 25 measurements.

### Bacterial cell growth conditions

Gram-negative *E. coli* (K12 strain) (BEI Resources, Virginia, USA) and Gram-positive *S. aureus* (RN0450 strain) (BEI Resources, Virginia, USA) bacteria were used to investigate the bactericidal activity of black Ti surfaces produced using the following processing parameters: *P*_*ICP* = _400 W, *P*_*RF*_ = 100 W, *p* = 0.02 mbar, *T = *20 °C, Δ*t* = 20 min, Cl_2_ pressure = 30 sccm, and Ar pressure = 2.5 sccm. Pre-cultures of *E. coli* and *S. aureus* were prepared by inoculating bacteria, previously grown on Lysogeny broth (LB) agar plate (BD Life Sciences, California, USA), and brain heart infusion (BHI) (Sigma-Aldrich, Missouri, USA) agar plates, in 100 ml of autoclaved LB and BHI broths, respectively, at 140 rpm and 37 °C. The bacterial cells were collected at their logarithmic stage of growth and their optical density at a wavelength of 600 nm (OD_600_) in the medium solution was adjusted to a value of 0.1. All sterile handling was performed in a laminar flow cabinet (Clean Air).

The polished and ICP RIE treated surfaces were cleaned with 70% ethanol, air dried, and exposed to UV light for 20 min prior to bacterial cell culture. For each bacterial cell type, triplicates of polished titanium surfaces (as the control group) and etched titanium surfaces (as the study group) were inoculated with 1 ml of bacterial suspensions in 24 well plates. The samples were then incubated at 37 °C for 18 hr. For SEM imaging, the adhered bacteria were fixated on the surfaces using a phosphate buffer saline solution (PBS) containing 4% formaldehyde (Sigma-Aldrich, Missouri, USA) and 1% glutaraldehyde (Sigma-Aldrich, Missouri, USA), and were subsequently washed with MilliQ water and 50%, 70%, and 96% ethanol, respectively. Finally, they were soaked in hexamethyldisilazane (Sigma-Aldrich, Missouri, USA) for 30 min and then air-dried. The specimens were then coated with a thin layer of gold for SEM imaging. Top views at low and high magnifications were acquired for investigating the killing efficiency of the surfaces. Bacteria with a totally disrupted cell wall or with irregular morphologies, were considered as dead/damaged bacteria^4,62^.

### Quantification of bactericidal activity

To quantify the bactericidal efficiency of the surfaces, live/dead staining of the adhered bacteria was performed using L7012 Live/Dead *Bac*Light Bacterial Viability Kit (Invitrogen) after 18 h of culture according to the manufacturer’s instructions. Briefly, the bacteria were stained by a 1:1 mixture of SYTO 9 green-fluorescent and propidium iodide red-fluorescent stains for 15 minutes incubated at room temperature. This method distinguishes between the live and dead bacteria based on the membrane integrity so the green dye penetrates both live and dead bacteria while the red dye only penetrates the bacteria with a damaged membrane. Following the staining, images were acquired using a Luca R 604 widefield fluorescence microscope (Andor Technology, UK) with 20x magnification from five different areas of each sample. The number of live and dead bacteria were counted using ImageJ software (NIH, US). The killing percentage was obtained by dividing the number of dead bacteria by the total number of attached bacterial cells. The images were set on threshold for red and green channels in ImageJ software (NIH, US) and the number of dead and live bacteria was counted using Analyse Particles feature of the software.

### Cytocompatibility of ICP RIE surfaces

Ti surfaces produced under similar conditions with those used for bacterial culture (*P*_*ICP*_ = 400 W, *P*_*RF*_ = 100 W, *p* = 0.02 mbar, *T* = 20 °C, Δ*t* = 20 min, Cl_2_ pressure = 30 sccm, and Ar pressure = 2.5 sccm) were cultured with murine preosteoblast cells (MC3T3-E1). Therefore, the cells were seeded on the samples in a 24 well-plate (Greiner Bio-One, NL) with a density of 20,000 cells per well (n = 3). After 1 day of culture in α-MEM (Thermo Fisher, US) the cells were fixed and stained for actin filaments and nuclei. Briefly, the cells were permeabilized with 0.5% Triton/PBS (Sigma-Aldrich, US) at 4 °C. Rhodamine phalloidin (Thermo Fisher, US) was diluted in 1% BSA/PBS solution (Sigma-Aldrich, US) (1:1000) and used for actin staining by incubation for 1 hour at 37 °C. Samples were then washed with 0.5% Tween/PBS (Sigma-Aldrich, US) and mounted on glass slides along with Prolong gold antifade reagent containing DAPI (Thermo Fisher, US) prior to imaging using a ZOE™ fluorescent cell imager (Bio-Rad, NL).

### Statistical analysis

To assess if there is a significant difference between the killing efficiency of the black Ti nanostructures and the polished titanium surface, an analysis of variance was conducted using the two-way ANOVA test followed by Tukey’s multiple comparisons test. The statistical analysis was conducted using GraphPad Prism 8 Software and a *p-*value less than 0.05 was considered to be statistically significant.

## Conclusions

In this study, a comprehensive research was performed to assess the effects of the main ICP RIE process parameters on the characteristics of the resulting nanostructures on titanium. By systematically changing the ICP RIE parameters, nanostructures with different shapes, lengths, diameters, surface wettability and mechanical properties could be produced.

The etched morphologies included porous structures and nanopillars with various diameters (26–76 nm), lengths (0.5–5.2 µm), and extent of clustering. The length and shape of the nanopillars were the most sensitive characteristics to the ICP RIE process conditions. Contact angle measurements showed that Ti ICP RIE nanostructures were hydrophilic with water contact angles between 7° ± 2° and 57° ± 5°. The pressure of the chamber was found to have a major impact on the specifications of the resulting nanostructures. The nanostructures produced at higher chamber pressures (*i.e*. 0.04 mbar) were shorter and were densely connected to each other, while the nanopillars were taller and more separated, and with a larger interspacing, when a chamber pressure of 0.005 mbar was used. The dense Ti nanostructures at 0.04 mbar enhanced the mechanical properties and resulted in higher values of the elastic modulus and hardness. Furthermore, the preliminary biological tests with bacterial and preosteoblast cells indicated bactericidal potential and non-cytotoxic effects, respectively. Therefore, the ICP RIE process is considered a highly promising route for fabrication of titanium bone implants with dual biofunctionalities.

## Supplementary information

supplementary information
