# Supplementary material for: Reactive ion etching for fabrication of biofunctional titanium nanostructures
Source: Sci Rep. 2019 Dec 11;9:18815. doi: 10.1038/s41598-019-55093-y (PMC6906493; doi:10.1038/s41598-019-55093-y)
Supplement: Supplementary file 1 — supplementary information [file 41598_2019_55093_MOESM1_ESM.pdf]

Reactive ion etching for fabrication of biofunctional titanium nanostructures

Mahya Ganjian[1*§], *Khashayar Modaresifar*[1], *Hongzhi Zhang*[2], *Peter-Leon Hagedoorn*[3], *Lidy E. Fratila-Apachitei*[1], *Amir A. Zadpoor*[1]

§ First author
* Corresponding author, email: m.ganjian@tudelft.nl

[1]Department of Biomechanical Engineering, Faculty of Mechanical, Maritime, and Materials Engineering, Delft University of Technology, Mekelweg 2, 2628CD, Delft, The Netherlands

[2]Department of Materials, Mechanics, Management & Design, Faculty of Civil Engineering and Geosciences, Delft University of Technology, Stevinweg 1, 2628 CN, Delft, The Netherlands

[3]Department of Biotechnology, Faculty of Applied Sciences, Delft University of Technology, Van der Maasweg 9, 2629HZ, Delft, The Netherlands

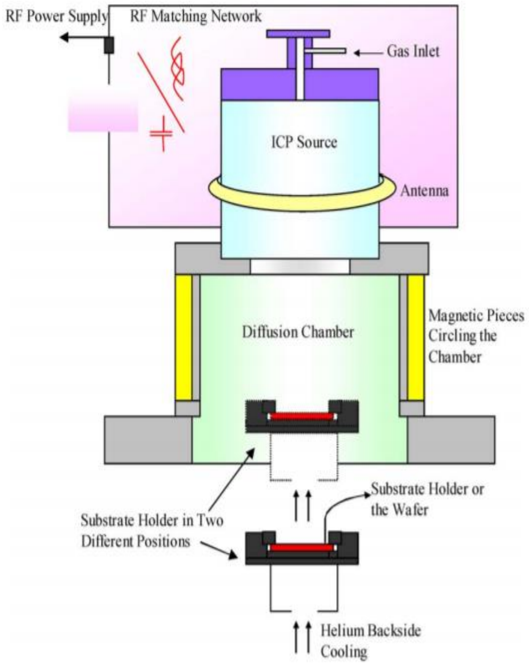

Figure S1. Schematics of ICP RIE setup. 8 Torr He gas was used as the backside cooling gas. reprinted from [1], copyright (2006), with permission from Journal of Non-crystalline Solids, published by Elsevier.

In ICP RIE, P_{RF} is used to control ion energy and self generated DC bias voltage is responsible for acceleration energy (Figure S1). These two parameters have an influence on each other and it is not possible to have control on both individually [2]. Moreover, P_{ICP} controls the ion flux. The relation between three above mentioned parameters is described by the following equation:

$$\mathbf{P_{RF}\ (W) \approx V_{DC}(V) \times P_{ICP}\ (W)} \quad (1)$$

Table S1. Characteristics of black Ti nanostructures under different ICP RIE conditions. ICP RIE condition: P_{ICP} 600 W , P_{RF} 100 W, Δt 20 min, p 0.02 mbar, T 20 °C, Cl_2 30 sccm, and Ar 2.5 sccm is called the reference.

ICP RIE condition	Length (µm)	Struts (nm)	Nanopillar diameter (nm)	θ_{H2O} (°)
titanium control surface	--	--	--	70.4 ± 1.8
reference sample	1.4 ± 0.2	27.4 ± 8.0	--	16.1 ± 1.4
Δt = 1 min	--	73.1 ± 16.9	--	57.5 ± 4.6
Δt = 5 min	0.5 ± 0.1	25.2 ± 7.5	--	22.2 ± 4.8
P_{RF} = 200 W	1.7 ± 0.1	17.7 ± 4.2	--	11.0 ± 1.7
P_{RF} = 300 W	3.8 ± 0.4	--	26.4 ± 7.0	7.0 ± 2.2
P_{ICP} = 200 W	0.7 ± 0.3	18.8 ± 6.2	--	31.3 ± 5.0
P_{ICP} = 400 W	1.1 ± 0.2	19.02 ± 4.9	--	27.1 ± 6.2
P_{ICP} = 800 W	1.9 ± 0.1	22.9 ± 7.0	28.1 ± 6.5	15.8 ± 5.2
p = 0.005 mbar	5.2 ± 0.3	--	76.0 ± 24.4	8.6 ± 2.1
p = 0.04 mbar	0.8 ± 0.1	19.1 ± 4.9	--	19.3 ± 5.6
T = 0 °C	0.6 ± 0.1	25.5 ± 6.1	--	47.1 ± 4.0
T = 40 °C	2.3 ± 0.3	14.8 ± 4.8	28.4 ± 7.4	26.9 ± 4.0
T = 60 °C	4.2 ± 0.6	--	71.5 ± 27.9	15.0 ± 3.0
Cl_2 = 10 sccm	1.2 ± 0.2	20.1 ± 4.8	--	21.5 ± 4.8
Cl_2 = 50 sccm	3.7 ± 0.1	--	32.0 ± 9.3	16.5 ± 3.3
Ar = 0 sccm	1.7 ± 0.3	20.8 ± 7.5	27.8 ± 8.6	15.9 ± 2.1
Ar = 5 sccm	3.9 ± 0.4	--	52.6 ± 12.3	7.5 ± 3.9

Ar = 10 sccm	3.9 ± 0.2	--	51.5 ± 12.3	7.7 ± 3.1

References

1 Goyal, A., Hood, V. & Tadigadapa, S. High speed anisotropic etching of Pyrex® for microsystems applications. *Journal of non-crystalline solids* **352**, 657-663 (2006).

2 Zhao, Y. *et al.* Controllable process of nanostructured GaN by maskless inductively coupled plasma (ICP) etching. *Journal of Micromechanics and Microengineering* **27**, 115004 (2017).

3 Hasan, J., Jain, S. & Chatterjee, K. Nanoscale Topography on Black Titanium Imparts Multi-biofunctional Properties for Orthopedic Applications. *Sci Rep* **7**, 41118, doi:10.1038/srep41118 (2017).
